# REASONS FOR RELOCATING TO ASSISTED LIVING: THE PUSH, THE PULL, AND DECISIONAL CONTROL

**DOI:** 10.1093/geroni/igz038.2375

**Published:** 2019-11-08

**Authors:** Evan Plys

**Affiliations:** University of Colorado School of Medicine, Denver, Colorado, United States

## Abstract

Using the push-pull framework, this study describes reasons for relocation and self-reported decisional control in the move to assisted living (AL). A sample of 202 residents of 21 ALs responded to a semi-structured questionnaire regarding their relocation. Participants most commonly relocated from a private local residence (n = 80, 40%), hospital/rehab facility (n = 27, 13%), or private long-distance residence (n = 24, 12%). The most frequently reported pull reasons to relocate to an AL were: “security and safety” (n = 46), “closer to family or friends” (n = 43), and “appearance of the facility” (n = 40). The most frequently reported push reasons to relocate from a previous residence were: “health problems” (n = 94), “others planned the move” (n = 87), and “fear of an accident” (n = 53). On average, participants who moved from other ALs reported the most decisional control in the move (M = 3.94, SD = 1.47), while participants from hospitals/rehab facilities reported the lowest control (M = 2.48, SD = 1.42). On average, participants who relocated from other ALs reported the most pull factors (M = 2.67, SD = 1.15), while participants from independent living communities reported the most push factors (M = 2.53, SD = 1.46). Results suggest that current residents commonly cite safety as both a push and pull reason for relocating to their AL. In addition, reasons for relocation and decisional control varied based on previous residence, which may be useful for identifying AL residents at risk for relocation stress.

